# Relationships between uptake of [^68^Ga]Ga-DOTA-TATE and absorbed dose in [^177^Lu]Lu-DOTA-TATE therapy

**DOI:** 10.1186/s13550-022-00947-2

**Published:** 2022-12-19

**Authors:** Anna Stenvall, Johan Gustafsson, Erik Larsson, Daniel Roth, Anna Sundlöv, Lena Jönsson, Cecilia Hindorf, Tomas Ohlsson, Katarina Sjögreen Gleisner

**Affiliations:** 1 Medical Radiation Physics, Lund, Lund University, Lund, Sweden; 2 Radiation Physics, Skåne University Hospital, Lund, Sweden; 3 Division of Oncology, Department of Clinical Sciences, Lund, Lund University, Lund, Sweden; 4 Department of Radiation Physics and Nuclear Medicine, Karolinska University Hospital, Stockholm, Sweden

**Keywords:** Radionuclide therapy, Dosimetry, Neuroendocrine tumours, Peptide receptor radionuclide therapy, Somatostatin receptor imaging, DOTA-TATE, ^68^Ga PET, ^177^Lu SPECT

## Abstract

**Background:**

Somatostatin receptor ^68^Ga PET imaging is standard for evaluation of a patient’s suitability for ^177^Lu peptide receptor radionuclide therapy of neuroendocrine tumours (NETs). The ^68^Ga PET serves to ensure sufficient somatostatin receptor expression, commonly evaluated qualitatively. The aim of this study is to investigate the quantitative relationships between uptake in ^68^Ga PET and absorbed doses in ^177^Lu therapy.

**Method:**

Eighteen patients underwent [^68^Ga]Ga-DOTA-TATE PET imaging within 20 weeks prior to their first cycle of [^177^Lu]Lu-DOTA-TATE. Absorbed doses for therapy were estimated for tumours, kidney, spleen, and normal liver parenchyma using a hybrid SPECT/CT–planar method. Gallium-68 activity concentrations were retrieved from PET images and also used to calculate SUVs and normalized SUVs, using blood and tissue for normalization. The ^68^Ga activity concentrations per injected activity, SUVs, and normalized SUVs were compared with ^177^Lu activity concentrations 1 d post-injection and ^177^Lu absorbed doses. For tumours, for which there was a variable number per patient, both inter- and intra-patient correlations were analysed. Furthermore, the prediction of ^177^Lu tumour absorbed doses based on a combination of tumour-specific ^68^Ga activity concentrations and group-based estimates of the effective half-lives for grade 1 and 2 NETs was explored.

**Results:**

For normal organs, only spleen showed a significant correlation between the ^68^Ga activity concentration and ^177^Lu absorbed dose (*r* = 0.6). For tumours, significant, but moderate, correlations were obtained, with respect to both inter-patient (*r* = 0.7) and intra-patient (*r* = 0.45) analyses. The correlations to absorbed doses did not improve when using ^68^Ga SUVs or normalized SUVs. The relationship between activity uptakes for ^68^Ga PET and ^177^Lu SPECT was stronger, with correlation coefficients *r* = 0.8 for both inter- and intra-patient analyses. The ^177^Lu absorbed dose to tumour could be predicted from the ^68^Ga activity concentrations with a 95% coverage interval of − 65% to 248%.

**Conclusions:**

On a group level, a high uptake of [^68^Ga]Ga-DOTA-TATE is associated with high absorbed doses at ^177^Lu-DOTA-TATE therapy, but the relationship has a limited potential with respect to individual absorbed dose planning. Using SUV or SUV normalized to reference tissues do not improve correlations compared with using activity concentration per injected activity.

**Supplementary Information:**

The online version contains supplementary material available at 10.1186/s13550-022-00947-2.

## Background

Peptide receptor radionuclide therapy (PRRT) with [^177^Lu]Lu-DOTA-TATE for treatment of somatostatin receptor (SSTR) expressing neuroendocrine tumours (NETs) [1, 2] is typically preceded by SSTR-PET imaging using [^68^Ga]Ga-DOTA-TATE or -TOC to ensure adequate receptor expression [3, 4]. The use of a theragnostic approach with the same or similar peptides for imaging and therapy offers opportunities for therapy stratification, but there is today no consensus on the predictive value of ^68^Ga-SSTR-PET/CT imaging with respect to response, absorbed doses, or activity uptakes in tumours and normal organs for therapy. A number of studies have investigated the relationship between SSTR expression quantified from ^68^Ga-SSTR-PET/CT and the outcome of [^177^Lu]Lu-DOTA-TATE or -TOC therapy of NETs [5–10]. When examining such relationships, it is often implicitly assumed that a high tumour uptake in pre-therapeutic ^68^Ga-SSTR-PET/CT images also infers high tumour uptake and absorbed dose during ^177^Lu therapy.

To the best of our knowledge, there is to date only one study that made a direct, quantitative comparison of results from [^68^Ga]Ga-DOTA-TOC PET and absorbed doses delivered during [^177^Lu]Lu-DOTA-TOC therapy for NET patients [11]. In that study, tumour dosimetry was performed for 21 patients based on serial planar ^177^Lu imaging, and a statistically significant correlation (*r* = 0.7) was found between the ^68^Ga-SUV (SUV_mean_ or SUV_max_) and the ^177^Lu absorbed dose [11]. Furthermore, a few reports on similar radiopharmaceuticals or indications are available. Krebs et al. [12] reported on the treatment of 20 NET patients using a SSTR antagonist (^177^Lu-satoreotide tetraxetan) with pre-therapeutic ^68^Ga-imaging and ^177^Lu dosimetry based on a hybrid SPECT–planar method. Various quantitative parameters were analysed, including tumour-to-normal-tissue SUV ratios, and the highest correlation (*r* = 0.5) was found between ^68^Ga-SUV_peak_ and the ^177^Lu absorbed dose to lesions [12]. Hänscheid et al. [13] reported data from 11 patients treated for meningioma, where ^177^Lu dosimetry was performed with a hybrid SPECT–planar method. They found that the ^68^Ga-SUV_max_ correlated well with the ^177^Lu activity concentration 1 h after administration (*r* = 0.95), whilst the correlation to ^177^Lu absorbed dose was moderate (*r* = 0.76). For [^177^Lu]Lu-PSMA, pre-therapeutic ^68^Ga-PET/CT and ^177^Lu absorbed doses have also been compared, e.g. by Peters et al. [14].

Investigation of possible relationships between uptakes of ^68^Ga-SSTR-PET and absorbed doses in ^177^Lu PRRT can be made from different perspectives. In the above-mentioned studies, the relationship was approached on a population level, reflecting the overall relationship across patients. For metastatic disease, analyses can also be made across the tumours within individual patients, addressing the distribution of uptakes and absorbed doses, i.e. whether a higher uptake of [^68^Ga]Ga-DOTA-TATE in one tumour than another generally means that the absorbed dose is higher for that tumour in [^177^Lu]Lu-DOTA-TATE therapy. Thirdly, the question can be posed as an estimation problem, to understand whether and how well absorbed doses in ^177^Lu PRRT can be predicted from a pre-therapeutic ^68^Ga-SSTR-PET. This perspective is relevant with regards to personalized dose planning, where both tumours and normal organs need to be considered. The various perspectives need to be considered separately, as they require different methods for evaluation.

Studies that compared the activity uptakes in ^68^Ga-SSTR-PET/CT with the uptakes and absorbed doses in ^177^Lu-PRRT have mainly used different variants of SUV for evaluation of the ^68^Ga images. Besides SUV, different tumour-to-tissue ratios have been proposed, where reference tissues include the liver parenchyma, spleen, or blood [5, 15, 16]. Using SUV ratios is partly methodologically motivated, as this may partly mitigate the SUV dependence on factors such as reconstruction settings, the PET/CT system, and the accumulation time [17]. Another motivation is the pharmacokinetics, as demonstrated for 10 patients examined by dynamic [^68^Ga]Ga-DOTA-TATE and -TOC PET/CT, leading to the suggestion of using the tumour-to-blood SUV ratio [16, 18]. However, a simpler, and more fundamental parameter than SUV is the activity concentration. Although SUV is well established as a metric in diagnostics and patient selection from ^68^Ga-SSTR-PET/CT [3, 4], the reasons for using SUV are less evident when attempting to find a relationship to the therapeutic absorbed dose from ^177^Lu. Specifically, the inclusion of the patient's weight can be questioned (SUV = activity concentration × weight/injected activity), as the weight does not enter the calculation of the absorbed dose to tumours and organs.

The aim of this study was to investigate whether and how parameters derived from [^68^Ga]Ga-DOTA-TATE PET/CT relate to the uptake and absorbed doses delivered during [^177^Lu]Lu-DOTA-TATE therapy in NET patients. As basic property for [^68^Ga]Ga-DOTA-TATE quantification the activity concentration per administered activity is calculated, which is then complemented by different SUV-based metrices. For [^177^Lu]Lu-DOTA-TATE both the activity concentration and the absorbed dose per administered activity are considered. Furthermore, the possibility to predict ^177^Lu absorbed doses for tumours based on quantitative [^68^Ga]Ga-DOTA-TATE PET/CT images combined with population mean effective half-lives for [^177^Lu]Lu-DOTA-TATE, separated on grade-1 and grade-2 NETs, is studied. This study thus aims to complement and expand on earlier studies, using modern quantification methods, and analysing data for both organs and tumours, considering correlations as well as the ability of absorbed dose prediction.

## Methods

### Patient data

The patients included in this study are a subset of patients from the Iluminet trial [19], which was designed to study the safety and efficacy of dosimetry-based therapy with [^177^Lu]Lu-DOTA-TATE in patients with well-differentiated metastatic neuroendocrine tumours. The current data set consists of the Iluminet patients treated at Skåne University Hospital, Lund, who had performed a [^68^Ga]Ga-DOTA-TATE PET/CT a maximum of 20 weeks prior to the first cycle of [^177^Lu]Lu-DOTA-TATE. Originally, inclusion in the Iluminet trial was based on Octreoscan® uptake, wherefore not all patients had performed a PET/CT during the screening phase.

A total of 18 patients (10 males, 8 females) were eligible for analysis. The median age was 66.5 years (range 35.5 to 79.7 years). The primary tumour origin varied between patients: eleven patients had a small intestinal NET, three had a pancreatic NET, two colon NET, one lung NET, and one patient had a primary NET of unknown origin. The median Ki67-index was 3% (range 1% to 18%). Median time from [^68^Ga]Ga-DOTA-TATE PET/CT to the first cycle of [^177^Lu]Lu-DOTA-TATE was 5.1 weeks (range 0.3 to 18 weeks). Treatment with long-acting somatostatin analogues (SSA) was held four weeks before administration of [^177^Lu]Lu-DOTA-TATE. Data on the time interval between injection of [^68^Ga]Ga-DOTA-TATE and last SSA administration were available for 9 patients, and was on average 18 days.

### Activity preparation and administration

The labelling of [^68^Ga]Ga-DOTA-TATE was performed using an established technique described in Gålne et al. [20]. Patients were prescribed an activity per body weight of 2.5 MBq/kg and received a total activity of (0.17 ± 0.04) GBq (mean ± standard deviation). The injected amount of peptide was (14 ± 6) nmol (equivalent to (20 ± 8) µg), and the fraction of the DOTA-TATE molecules that were radiolabelled was $\left( {1.3 \pm 0.5} \right) \times 10^{ - 4}$. The radiochemical purity exceeded the lower limit of 91% for all administrations.

[^177^Lu]Lu-DOTA-TATE was prepared as previously described [21]. Patients were prescribed 7.4 GBq and received (7.45 ± 0.06) GBq. The injected amount of peptide was (133 ± 8) nmol ((190 ± 10) µg). The fraction of radiolabelled DOTA-TATE molecules was $\left( {7.7 \pm 0.4} \right) \times 10^{ - 2}$. The radiochemical purity exceeded the lower limit of 95% for all administrations. Kidney-protective amino acids were co-administered (2 L VAMIN® 14 g N/L) over 8 h, with 125 mL administered before the approximately 30 min long administration of DOTA-TATE. A corresponding co-infusion was not given for the [^68^Ga]Ga-DOTA-TATE administration.

All activity meters used in this study (Capintec CRC-15, Capintec CRC-55tR, Comecer Vik-202) were calibrated with traceability to primary standard for ^177^Lu, ^68^Ga, and ^18^F.

### Image data

#### ^68^Ga PET imaging

PET/CT acquisitions were performed on a GE Discovery PET/CT 690. The time between injection and imaging was (64 ± 5) min. Images were acquired from head to mid-thigh, with acquisition time 3 min per bed position. Tomographic images were reconstructed with an in-plane matrix size of 192 × 192 and voxel size 3.65 × 3.65 × 3.27 mm^3^, using time-of-flight information and OS-EM with 3 iterations and 12 subsets, compensation for attenuation and scatter, three-dimensional point-spread function (PSF) modelling (referred to as VPFX-S on the camera system), a transaxial 5 mm full width at half maximum (FWHM) Gaussian post-filter, and an axial z-filter.

#### ^177^Lu SPECT imaging

SPECT/CT studies were acquired at nominally 1 d post-administration. For 17 patients the SPECT/CT was acquired (21.9 ± 1.1) h after administration, whilst one had the SPECT/CT performed at 94.6 h. To make SPECT-derived data consistent, the latter set of data were recalculated to the time point of the corresponding day one planar image (22.3 h) using the planar-derived effective half-lives for the respective tissues. The timing of the SPECT-derived data for all patients was then (21.9 ± 1.0) h after administration. Two systems were used, GE Discovery VH (1 patient) and GE Discovery 670 (17 patients). Both systems were equipped with medium-energy collimators and projections were acquired in 60 angles over 360^◦^ in 128 × 128 matrices with pixel sizes 4.02 × 4.02 mm^2^ (Discovery VH) or 4.42 × 4.42 mm^2^ (Discovery 670). An energy-window centred at 208 keV with a width of 20% (Discovery VH) or 15% (Discovery 670) was employed. Tomographic images were reconstructed using an off-line program using OS-EM with 10 subsets, including compensations for attenuation and scatter using the model-based ESSE scatter-compensation method [22]. Since different steps in the image-based dosimetry method required SPECT images with different properties, three different reconstruction settings were used [23]. Briefly, the first type of SPECT image (ASR-8) was used for visual inspection and manual delineation of organs, and was reconstructed with resolution compensation and 8 iterations. The second type of SPECT image (AS-8) was used for automatic delineation of tumours, and was reconstructed without resolution compensation with 8 iterations. The third type of SPECT image (ASR-40) was used for activity and absorbed dose estimation, and was reconstructed with resolution compensation and 40 iterations.

#### ^177^Lu planar gamma camera imaging

Dosimetry for ^177^Lu was performed using a hybrid SPECT–planar approach. For this purpose, planar whole-body gamma camera images were acquired at nominally 1 h, 24 h, 96 h, and 168 h post-injection (p.i.), using the same camera systems as for SPECT/CT. For each time point, anterior–posterior scans were co-registered to a scout radiograph and the geometric mean calculated on a pixel-by-pixel basis. Attenuation and scatter correction was performed using the scout radiograph to estimate the attenuation and scatter depth. This process yielded whole-body planar images with pixel values in projected activity [24].

#### Camera calibration

The PET camera system was calibrated for ^18^F against the activity meter once every three months, and ^18^F SUV verification measurements were made at least once per month. Retrospectively, SUV measurements were also made for ^68^Ga. The SUV for ^18^F was obtained to (0.99 ± 0.03) g mL^−1^, while for ^68^Ga it was (0.94 ± 0.02) g mL^−1^ A similar systematic SUV deviation from 1.00 g mL^−1^ for ^68^Ga has been reported by others [25, 26]. In this work, the observed deviation was considered in the PET image-based quantification by division of the activity concentrations from volumes of interest (VOIs) by the factor 0.94. The gamma camera was calibrated for ^177^Lu by measurement of the system sensitivity in air [27], which was used for both planar and SPECT image calibration.

### Quantification of the activity concentration

For both PET and SPECT images, VOIs were delineated over organs and tumours and recovery coefficients (RCs) applied, as described below. Activity concentrations were calculated as the total activity in the VOI divided by the VOI volume (SPECT) or the mean value in the VOI (PET), divided by the relevant RC. All data were normalized to the injected activity and decay-corrected to the time of administration using the physical half-lives of ^68^Ga or ^177^Lu [28, 29], giving the activity concentration per injected activity, henceforth referred to as AC/IA. SUV values (SUV_max_ and SUV_mean_) were calculated according to clinical practice, i.e. based on non-partial-volume corrected activity concentrations, decay-corrected to the time of injection, normalized to the injected activity, and multiplied by the body weight. For SUV_max_ the maximum voxel value in the VOI was used.

### Image segmentation

#### Organ delineation

For left and right kidney, liver parenchyma, and spleen VOIs were manually defined in the SPECT/CT and PET/CT images. For spleen and kidneys, whole-organ VOIs were defined mainly using the CT for guidance, although, in case of misalignment between the CT and SPECT or PET, the VOIs were adjusted. For liver parenchyma, multiple small VOIs were defined with the ambition to avoid tumour-infiltrated liver. For PET/CT images, the blood activity concentration was quantified by placing a small VOI in 10 consecutive transverse planes in the descending aorta, taking care to avoid regions close to lesions or lymph nodes with high activity uptakes. For planar images, small regions of interest were drawn centrally in the respective organ, with a margin to the organ contour to avoid interference from activity in neighbouring tissues.

#### Tumour delineation

To be eligible for assessment, a given tumour had to be well identifiable in both PET and SPECT images. To be suitable for hybrid planar–SPECT/CT time-activity analyses a further requirement was limited signal overlap from activity in surrounding tissues, and a set of criteria for tumour inclusion, detailed in Roth et al. [23, 30], were applied.

For planar images, tumour delineation was performed using a semiautomatic active rays-based technique [30]. For SPECT and PET images, a semiautomatic method based on Fourier surfaces was applied [31]. Tumours were manually identified by defining a rough VOI around the tumour with a margin. For SPECT, the ASR-8 images were used for manual selection and the Fourier surface method was then applied on the AS-8 images. For PET images, the clinical reconstructions were used for both manual identification and the subsequent automatic delineation. A few delineations were modified after review by the responsible oncologist.

The Fourier surface method has been previously validated for tumour segmentation in ^177^Lu SPECT images by Gustafsson et al. [31], where it was found that reconstruction using AS-8, i.e. without resolution recovery, gave good performance in terms of volume preservation. To evaluate the performance for the PET images from the camera system used in this work, experimental data from Jönsson et al. [32] were used. These included PET/CT images of six ^68^Ga-filled spheres in a NEMA IEC Body Phantom with volumes between 0.52 mL and 26.5 mL, and background-to-sphere activity concentration ratios of 0%, 20%, 40%, 60%, and 80%. At application of the Fourier surface segmentation method to these images, a systematic negative bias in the volumes was obtained, likely as a result of the resolution recovery included in the reconstruction. To correct for this volume error, the physical sphere volume $V_{{\text{p}}}$ was mapped to the volume estimated from segmentation, $V_{{\text{s}}}$, and the background-to-object activity concentration ratio, $\eta$, following

1 $$V_{{\text{p}}} = a_{0} + a_{1} V_{{\text{s}}} + a_{2} \eta + a_{3} V_{{\text{s}}} \eta ,$$

where $a_{0}$, $a_{1}$, $a_{2}$, and $a_{3}$ are parameters determined through linear regression. At application for determination of tumour volumes and activity quantification from patient PET images, Eq. 1 was used as a post-segmentation correction, such that $V_{{\text{s}}}$ and $\eta$ was determined from the images, and the tumour volume obtained as $V_{{\text{p}}}$, as described in Appendix.

### Partial-volume correction

As the general blood activity concentration differed substantially between 1 h and 24 h after DOTA-TATE administration, the image contrast and general background level differed between the ^68^Ga-PET and ^177^Lu-SPECT images. Moreover, the spatial resolution of the two modalities differed. For these reasons, different strategies were required for partial-volume correction (PVC).

#### PVC of organ data

For SPECT, kidneys and spleen were corrected for spill-out using an RC of 0.85, as previously determined for kidneys and spleen [27, 33]. The RC applied for liver parenchyma was unity, since the VOIs used were substantially smaller than the organ extension.

For PET, the RCs for kidneys and spleen were determined for each separate VOI, by convolving the VOI mask with the PSF of the reconstructed images. The PSF was determined using matched filter analysis [34] applied to the ^68^Ga sphere phantom data described above. The FWHM of the Gaussian PSF was determined to 6.4 mm (isotropic). The RC for liver parenchyma was unity.

#### PVC of tumour data

For ^177^Lu-SPECT, compensation for spill-out of object signal was made using a previously reported expression of the RC as a function of volume, $R\left( {V_{{\text{p}}} } \right)$ following

2 $$R\left( {V_{{\text{p}}} } \right) = \frac{1}{{1 + \left( {\frac{\alpha }{{V_{{\text{p}}} }}} \right)^{\beta } }},$$

where $\alpha$ and $\beta$ are two fitting parameters [23, 31, 35]. These parameters were determined based on sphere phantom experiments with $V_{{\text{p}}}$ representing the physical sphere volumes [23]. At application of Eq. 2 for PVC and activity quantification of patient tumours, the volumes obtained from the Fourier surface segmentation were applied [31].

For the ^68^Ga-PET images, with a comparably high blood background level, both the spill-out of object signal and spill-in from background were considered. The ^68^Ga sphere phantom data from Jönsson et al. described above were used to establish the recovery for the camera system used. In these images, spherical VOIs were defined with volumes according to the physical sphere volumes, $V_{{\text{p}}}$. The recovery was calculated as the apparent activity concentration in the respective VOI, divided by the activity concentration from phantom preparation. The RC was parametrized according to

3 $$R\left( {V_{{\text{p}}} ,\eta } \right) = R_{0} \left( {V_{{\text{p}}} } \right) + \left[ {1 - R_{0} \left( {V_{{\text{p}}} } \right)} \right] \cdot f \cdot \eta + \left[ {1 - R_{0} \left( {V_{{\text{p}}} } \right)} \right] \cdot \left[ {1 - f} \right] \cdot \eta^{2} ,$$

where $R_{0} \left( {V_{{\text{p}}} } \right)$ is the RC expression in Eq. 2, $\eta$ is the background-to-object activity concentration ratio, and $f$ is a fitting parameter in the interval $f \in \left[ {0,1} \right]$. For a non-radioactive background then $R\left( {V_{{\text{p}}} ,0} \right) = R_{0} \left( {V_{{\text{p}}} } \right)$, i.e. the same expression as in Eq. 2. When the activity concentration in the object and background are identical then $R\left( {V_{{\text{p}}} ,1} \right) \equiv 1$. The values of $\alpha$, $\beta$ and $f$ were obtained by fitting Eq. 3 to the phantom data, using nonlinear least squares with Levenberg–Marquardt’s method [36]. The fitted function $R\left( {V_{{\text{p}}} ,\eta } \right)$ is shown in Appendix, where the application of Eq. 3 is also described.

### Absorbed dose calculation for ^177^Lu

The time-sequence of planar images were used to estimate the shape of the time-activity curves. Region-specific, relative activity values were calculated as the mean signal per pixel in the ROIs. For organs, background correction was applied by subtracting the mean signal in a ROI placed over the patient’s thigh, assumed to represent an unspecific, general body background. For tumours, the average of the five highest pixel values within the ROI was used [30]. Curve fitting of the activity versus time data was performed using unweighted nonlinear least squares. For organs, a mono-exponential function was fitted to the last three time points, and a linear function between the first and second time point. For tumours, the curve consisted of a quadratic function between the first two time points and a mono-exponential function for the last three time points [23, 30]. To calculate the absorbed dose, a Monte Carlo program based on the EGS4 code with PRESTA was used [37, 38]. Absorbed dose rate images were calculated using the ASR-40 SPECT/CT images as input. Each VOI was applied to the volume-averaged absorbed dose rate, which was then corrected using the relevant RC. Absorbed dose rate curves were obtained by rescaling the fitted time-activity curves to the absorbed dose rate, with a scaling factor determined from the curve value at the time of SPECT imaging. Finally, the absorbed dose was calculated by analytical integration of this rescaled curve. The assumption was thus made that the absorbed dose rate curves followed the time-activity curves [23]. For each of the segmented structures (organs and tumours), the absorbed dose per injected activity (AD/IA) was calculated.

### Prediction of ^177^Lu tumour absorbed doses from ^68^Ga PET images

The possibility to predict tumour absorbed doses for [^177^Lu]Lu-DOTA-TATE based on the [^68^Ga]Ga-DOTA-TATE activity concentrations was explored. Based on previously published patient data, the assumption was made that the time-activity curves followed a mono-exponential pattern, with effective half-lives of 103 h and 81 h for grade-1 and grade-2 NET patients, respectively [23]. The ^68^Ga activity concentration from images was propagated back to the concentration at time $t = 0$, and the corresponding ^177^Lu activity concentration calculated by scaling to the injected activities, $A_{{{\text{inj}},{ }177{\text{Lu}}}} /A_{{{\text{inj}},{ }68{\text{Ga}}}}$. The predicted ^177^Lu absorbed doses were then calculated by the assumption of electron local energy deposition [39], a tissue density of 1.04 g mL^−1^, and integration of the mono-exponential time-activity curves. The predicted ^177^Lu absorbed doses were compared with the absorbed doses measured at therapy.

### Statistical analysis

For organs (kidneys, spleen, and liver parenchyma), the connection between the uptakes of [^68^Ga]Ga-DOTA-TATE and the relevant dosimetric parameters for [^177^Lu]Lu-DOTA-TATE were studied using Pearson’s correlation coefficient. For kidneys, the mean of the data for left and right kidneys was considered to obtain independent data points.

For tumours, a cutoff volume was introduced, to exclude tumours with size close to the system spatial resolution [31]. Thus, tumours with volumes smaller than 5 mL as quantified from PET images were excluded from further analysis. Separate analyses were made of inter- and intra-patient correlations, using weighted correlation coefficients and repeated-measures correlations, respectively, as suggested by Bland and Altman [40, 41]. For the weighted correlation, each patient contributed with a single data point in the form of the mean, and the correlation was calculated using the number of tumours per patient as weights. For the repeated-measures correlation, all tumour data were used. The slope was assumed to be common for all patients, while the intercept was treated as patient-specific in the linear fitting. Correlations for which $p < 0.05$ were considered statistically significant.

To investigate the stability of the correlation coefficients, a leave-one-out analysis was also made in which single data points were removed and the correlation analysis repeated. The leave-one-out analyses were performed for organ correlations and inter-patient correlations for tumours.

The agreement between tumour absorbed doses predicted from ^68^Ga PET and those quantified from peri-therapeutic ^177^Lu images was studied using a Bland–Altman plot. Since the errors were expected to scale with absorbed dose, the relative deviations were studied rather than the absolute deviations. To achieve symmetry of positive and negative deviations, the analysis was performed using the logarithms of the ratios. The mean deviation and 95% coverage intervals were calculated for the logarithmized ratios which were then transformed back to linear relative deviations.

## Results

### Organ and tumour volumes

**Fig. 1:**
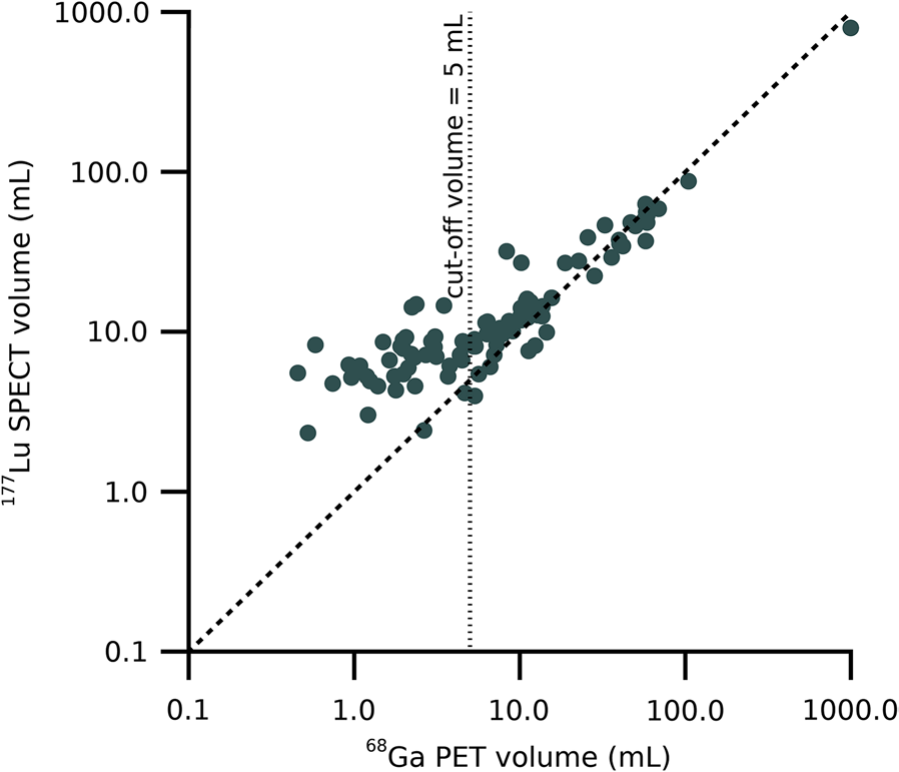
Tumour volumes estimated from ^68^Ga-PET and ^177^Lu-SPECT. The dashed line indicates the identity line, and the vertical dotted line indicates the volume cutoff of 5 mL employed in the analyses

For kidneys and spleen, the mean relative difference (± standard deviation) between organ volumes determined from ^177^Lu-SPECT and ^68^Ga-PET images were obtained to (2 ± 10) % and (-1 ± 12) %, respectively. Figure 1 shows the relationship between tumour volumes derived from ^177^Lu-SPECT and ^68^Ga-PET. Of the total 92 tumours delineated, only those with a PET-derived volume above 5 mL were included for further analysis (*n* = 52). For these tumours, the relative deviations between ^177^Lu-SPECT and ^68^Ga-PET volumes were (20 ± 52) %. For volumes below the cutoff volume, there was an increasingly larger systematic volume deviation, where most ^177^Lu-SPECT-derived volumes were larger than those derived from ^68^Ga-PET.

### Organ absorbed doses and activity concentrations

**Fig. 2:**
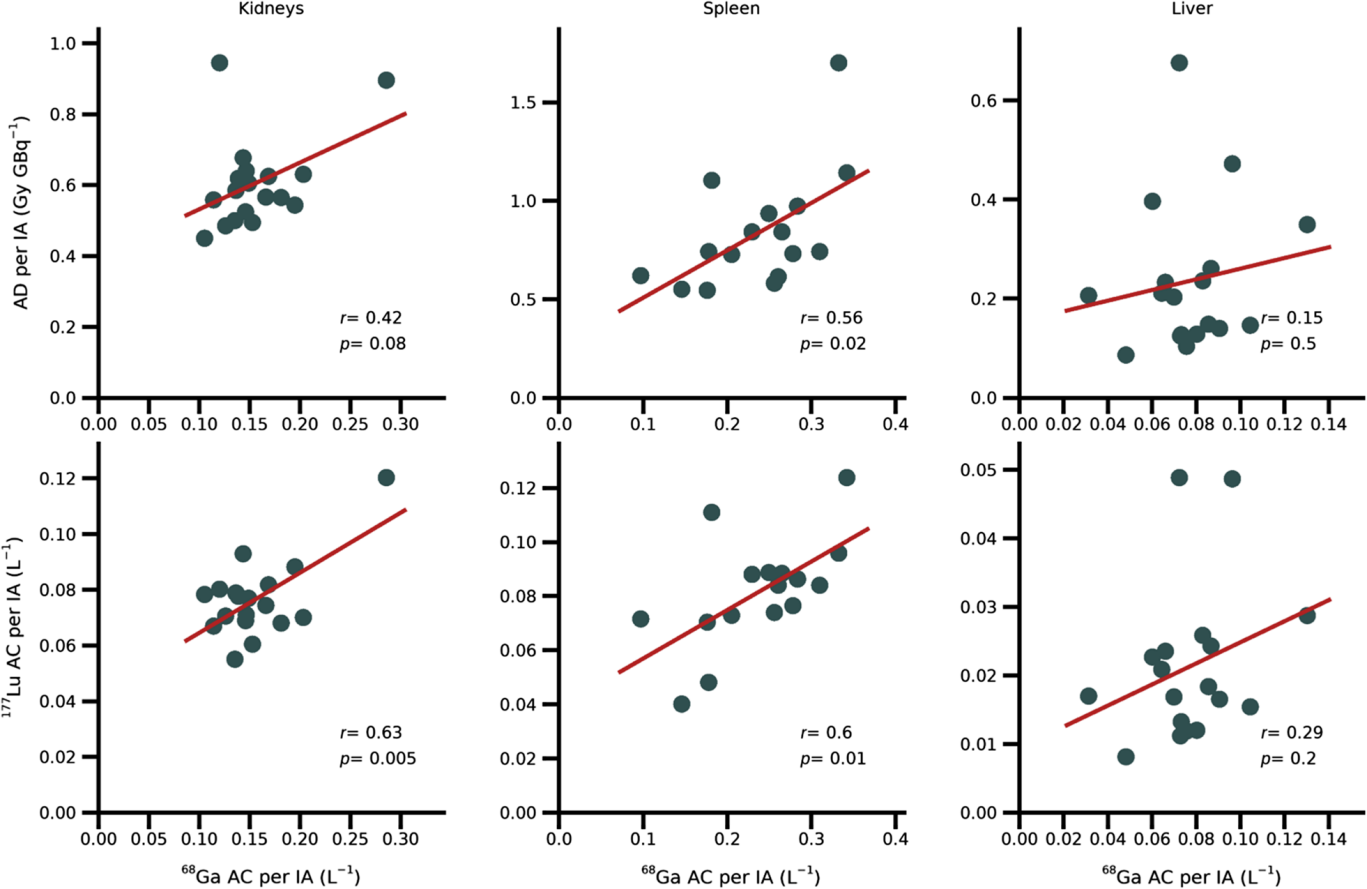
Observed relationship between [^68^Ga]Ga-DOTA-TATE and [^177^Lu]Lu-DOTA-TATE for organs. Lutetium-177 absorbed dose per injected activity (upper row) and ^177^Lu activity concentration per injected activity (lower row) are shown as a function of the ^68^Ga activity concentration per injected activity for kidneys (left column), spleen (middle), and liver (right)

Figure 2 shows results for kidneys, spleen, and liver, including the ^177^Lu absorbed dose (AD/IA) and activity concentration, ^177^Lu-AC/IA, both as a function of the ^68^Ga-AC/IA. The correlation coefficients, regression parameters, and intervals obtained for the correlation coefficient in the leave-one-out analysis (leave-one-out interval, LOOI) are summarized in Tables 1 and 2, where the correlation coefficients when using ^68^Ga SUV_mean_ as explanatory variable are also included.

**Table 1:** Results for organs of the ^177^Lu-AD/IA and ^177^Lu-AC/IA, as a function of two ^68^Ga-PET-derived metrices

	Kidneys				Spleen				Liver			
	*r*	*p*	*k*/*m*	LOOI	*r*	*p*	*k*/*m*	LOOI	*r*	*p*	*k*/*m*	LOOI
^*177*^*Lu-AD/IA*												
^68^Ga AC/IA	0.42	0.08	1.3/ 0.40	− 0.018; 0.76	0.56	0.02	2.4/0.27	0.46; 0.64	0.15	0.5	1.1/0.15	0.0; 0.28
^68^Ga SUV_mean_	0.081	0.7	0.0045/0.56	− 0.29; 0.62	0.63	0.008	0.030/0.35	0.37; 0.73	− 0.26	0.3	− 0.026/0.38	− 0.35; − 0.15
^*177*^*Lu-AC/IA*												
^68^Ga AC/IA	0.63	0.005	0.22/0.043	0.096; 0.70	0.60	0.01	0.18/0.039	0.49; 0.76	0.29	0.2	0.15/0.0094	0.20; 0.41
^68^Ga SUV_mean_	0.63	0.005	0.0038/0.039	0.42; 0.76	0.60	0.01	0.0020/0.050	0.47; 0.77	− 0.081	0.8	− 0.00062/0.025	− 0.22; 0.066

Correlation coefficients (*r*), p-value (*p*), coefficients for the linear equation (*y* = *kx* + *m*, presented as *k*/*m*) and leave-one-out interval (LOOI, min; max)

**Table 2:** Results for tumours of the ^177^Lu-AD/IA with respect to various PET-derived explanatory variables

	Inter-patient correlations				Intra-patient correlations		
	*r*	*p*	*k*/*m*	LOOI	*r*	*p*	*k*
^68^Ga AC/IA	0.71	0.004	6.0/1.5	0.62;0.81	0.44	0.04	3.0
^68^Ga SUV_mean_	0.63	0.02	0.13/1.1	0.53;0.77	0.47	0.02	0.071
^68^Ga SUV_max_	0.58	0.03	0.075/1.3	0.47;0.71	0.45	0.03	0.045
^68^Ga SUV_mean_/SUV_blood_	0.52	0.06	0.024/2.8	0.41;0.68	0.48	0.02	0.020
^68^Ga SUV_mean_/SUV_liver_	0.43	0.1	0.34/2.5	0.29;0.64	0.46	0.03	0.31
^68^Ga SUV_mean_/SUV_spleen_	0.27	0.4	0.60/2.9	0.13;0.45	0.19	0.5	0.55

Correlation coefficients (*r*), p-value (*p*), coefficients for the linear equation (*y* = *kx* + *m*, presented as *k*/*m*) and leave-one-out interval (LOOI, min; max)

For kidneys and spleen, there were significant (*p* < 0.05) positive correlations for the ^177^Lu-AC/IA with respect to both ^68^Ga-AC/IA and ^68^Ga SUV_mean_, but the LOOI for kidneys was large, indicating that the result was unstable. For the ^177^Lu-AD/IA, correlations were only significant for spleen. All significant correlations had approximately $r = 0.6$.

### Tumour absorbed doses and activity concentrations

**Fig. 3:**
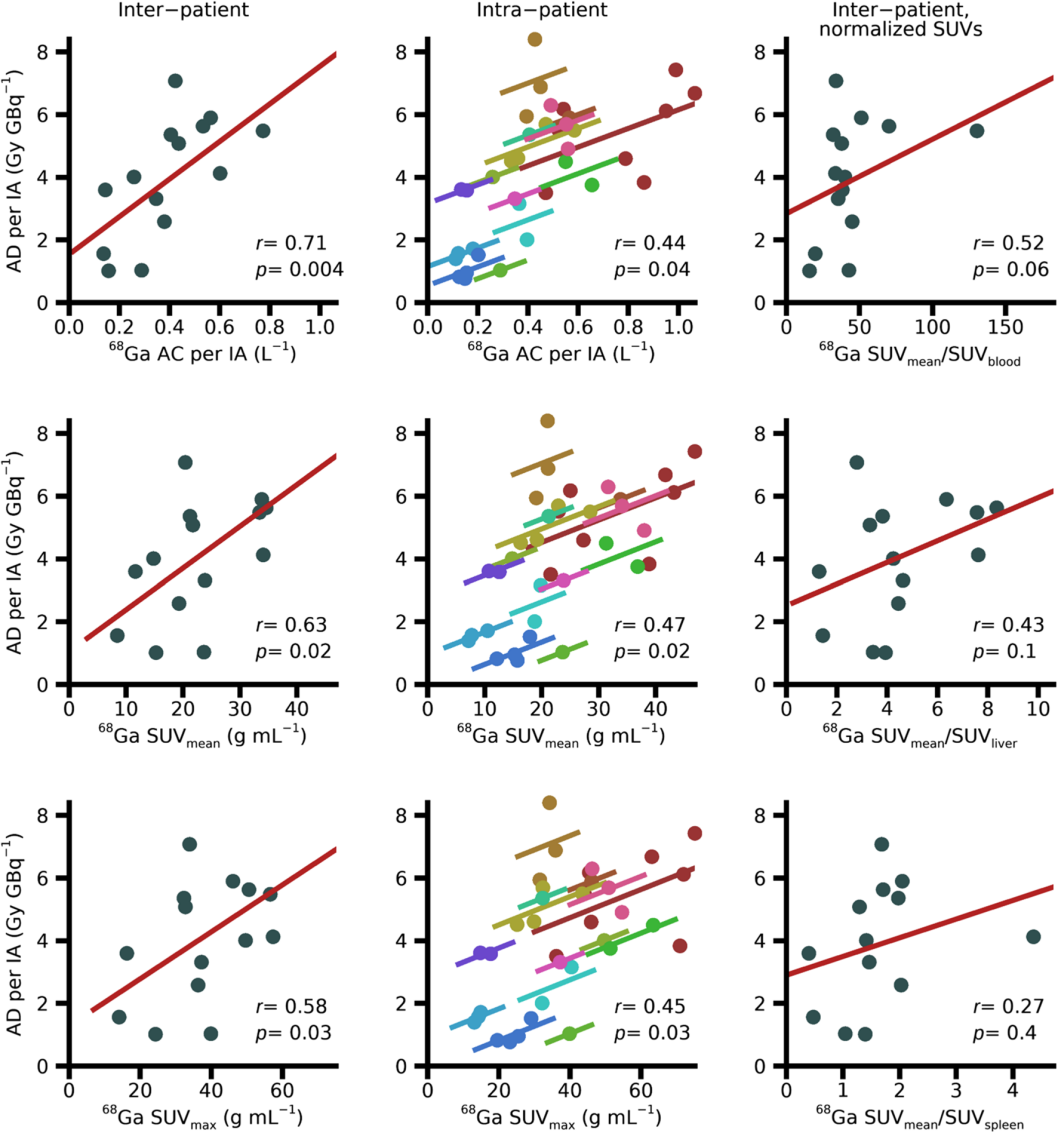
Observed relationship between [^68^Ga]Ga-DOTA-TATE and [^177^Lu]Lu-DOTA-TATE absorbed doses to tumours. Inter-patient (left and right columns) and intra-patient (middle column) analyses of the ^177^Lu-absorbed dose per injected activity are shown as a function of the ^68^Ga activity concentration per injected activity, ^68^Ga-SUV_mean_, ^68^Ga-SUV_max_, and various ^68^Ga-SUV ratios. Data underlying the inter-patient analyses are the means across the tumours in each patient, whereas the intra-patient analyses are based on data for the separate tumours in each patient, as indicated by the different colours

**Fig. 4:**
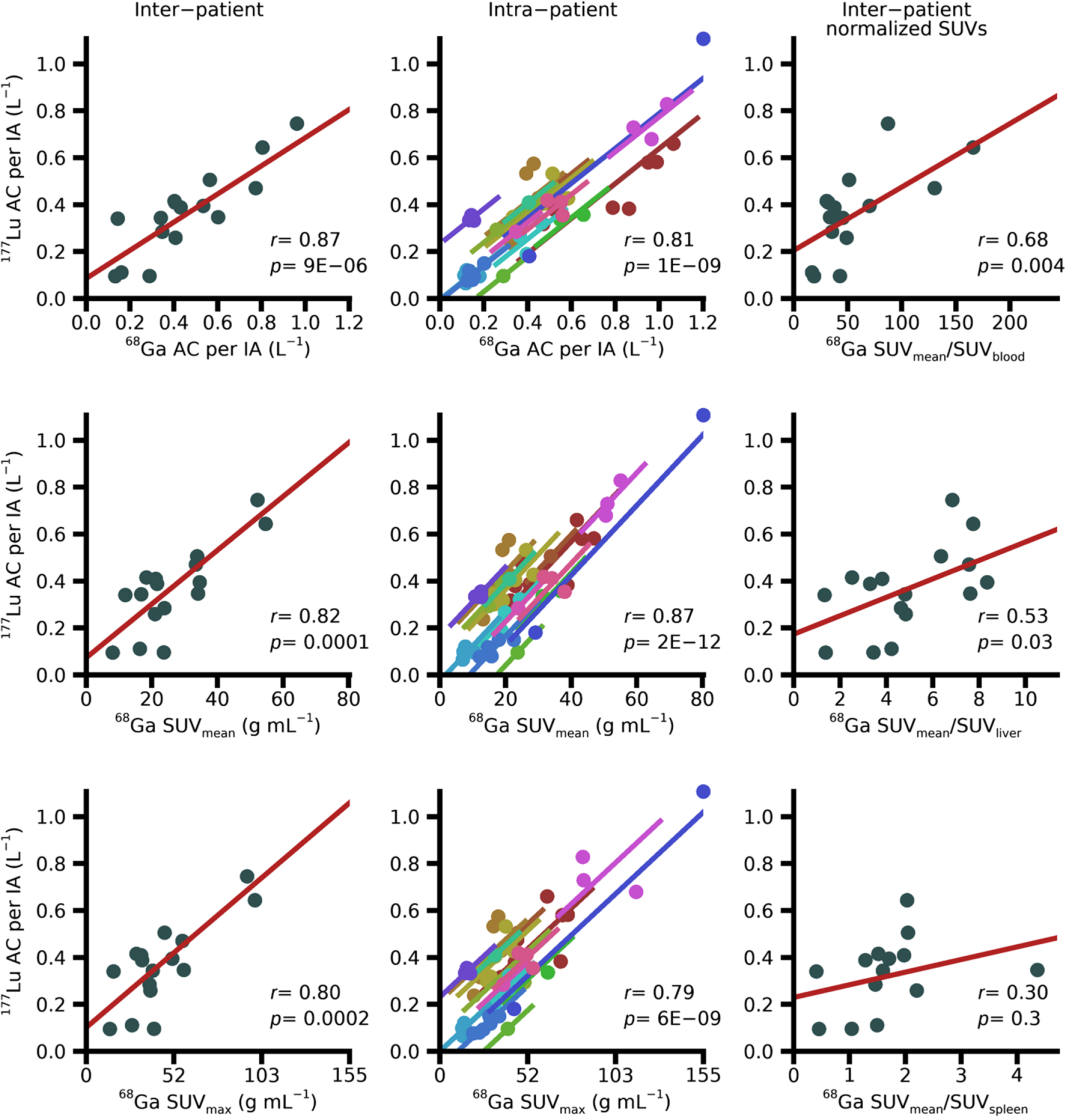
Observed relationship between [^68^Ga]Ga-DOTA-TATE and [^177^Lu]Lu-DOTA-TATE activity concentrations in tumours. Inter-patient (left and right columns) and intra-patient (middle column) analyses of the ^177^Lu activity concentration per injected activity are shown as a function of ^68^Ga activity concentration per injected activity, ^68^Ga-SUV_mean_, ^68^Ga-SUV_max_, and various ^68^Ga-SUV ratios. For the intra-patient analyses, different colours represent different patients

Figure 3 shows the ^177^Lu-AD/IA for tumours, as a function of the ^68^Ga-AC/IA, ^68^Ga-SUV_mean_, ^68^Ga-SUV_max_, and various ratios of ^68^Ga-SUV_mean_. For the latter, reference tissues were blood, liver parenchyma, and spleen. Relationships when using ^68^Ga-AC/IA, ^68^Ga-SUV_mean_, or ^68^Ga-SUV_max_ as explanatory variable are shown as both inter- and intra-patient correlations. The different SUV ratios are only shown on an inter-patient basis since the normalization is not expected to affect the intra-patient relationships. A summary of the obtained correlation coefficients, regression parameters, and LOOIs are given in Table 2. Figure 4 and Table 3 show corresponding results when the ^177^Lu-AC/IA is used as dependent variable.

**Table 3:** Results for tumours of the ^177^Lu-AC/IA with respect to various PET-derived explanatory variables

	Inter-patient correlations				Intra-patient correlations		
	*r*	*p*	*k*/*m*	LOOI	*r*	*p*	*k*
^68^Ga AC/IA	0.87	9 × 10^−6^	0.60/0.085	0.83;0.91	0.81	1 × 10^−9^	0.74
^68^Ga SUV_mean_	0.82	1 × 10^−4^	0.011/0.073	0.74;0.86	0.87	2 × 10^−12^	0.015
^68^Ga SUV_max_	0.80	2 × 10^−4^	0.0062/0.10	0.71;0.85	0.79	6 × 10^−9^	0.0068
^68^Ga SUV_mean_/SUV_blood_	0.68	0.004	0.0027/0.20	0.62;0.73	0.83	2 × 10^−10^	0.0044
^68^Ga SUV_mean_/SUV_liver_	0.53	0.03	0.039/0.17	0.42;0.62	0.79	8 × 10^−9^	0.075
^68^Ga SUV_mean_/SUV_spleen_	0.30	0.3	0.054/0.23	0.11;0.42	0.84	2 × 10^−8^	0.33

Correlation coefficients (*r*), p-values (*p*), coefficients for the linear equation (*y* = *kx* + *m*, presented as *k*/*m*) and leave-one-out interval (LOOI, min; max)

The inter-patient analyses showed significant correlations, exceptions being the ^177^Lu-AD/IA as a function of any of the ^68^Ga-SUV ratios, and the ^177^Lu-AC/IA as a function of SUV_mean_/SUV_spleen_. In general, the correlations were stronger for ^177^Lu-AC/IA than for ^177^Lu-AD/IA. Using the various SUV ratios as explanatory variables yielded weaker inter-patient correlations and did generally not improve the intra-patient correlations compared to when using ^68^Ga-AC/IA, SUV_mean_, or SUV_max_. The intra-patient analyses showed significant repeated-measures correlations, with the exception of ^177^Lu-AD/IA as a function of SUV_mean_/SUV_spleen_. Thus, within a given patient, the variation in ^68^Ga uptakes between tumours generally also translated to a difference in ^177^Lu uptakes and absorbed doses.

### Prediction of ^177^Lu tumour absorbed doses from ^68^Ga PET images

**Fig. 5:**
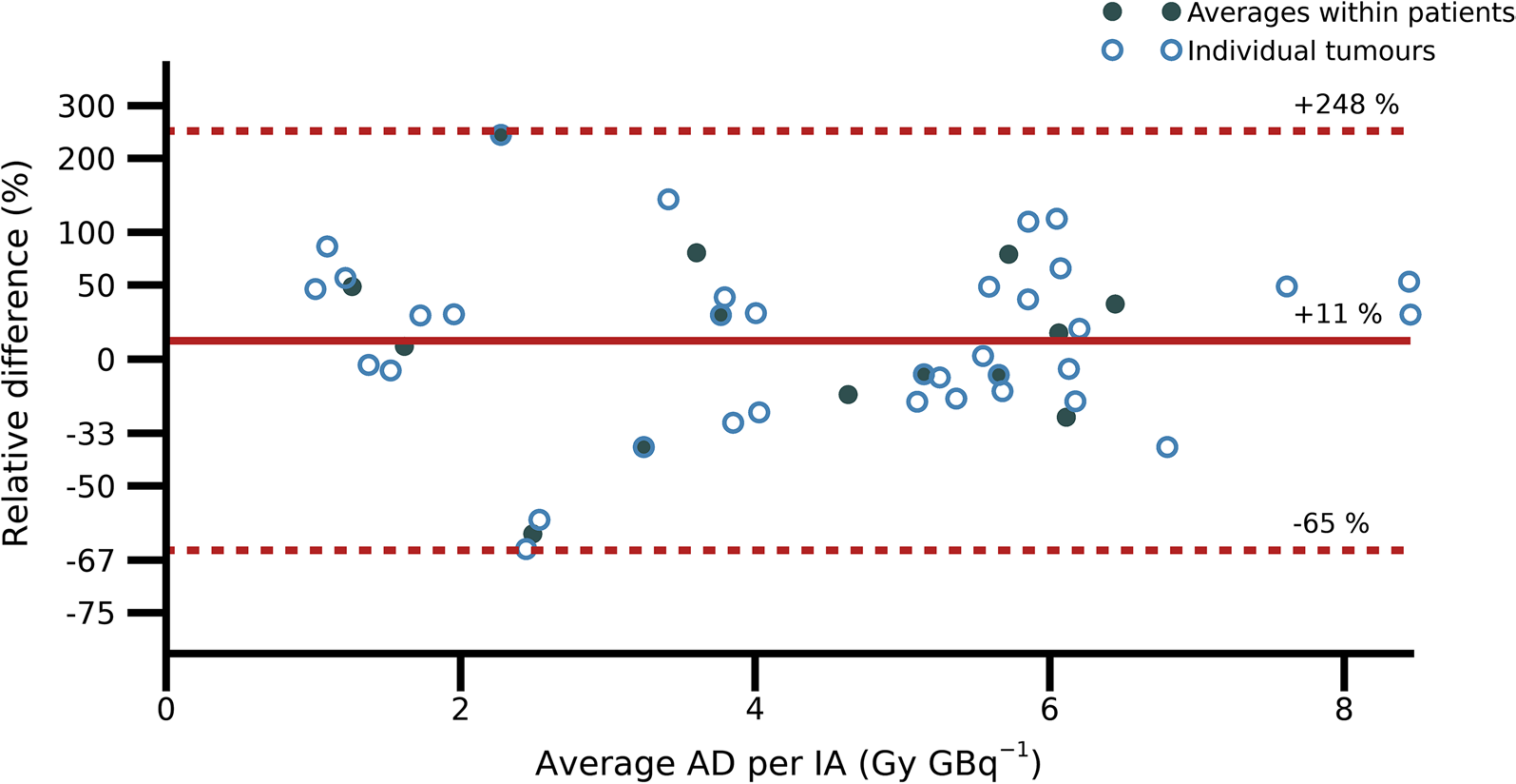
Bland–Altman plot of tumour absorbed doses estimated from ^68^Ga-PET images and ^177^Lu SPECT. Quantification of the mean deviation (solid line) and the 95% coverage interval (dashed lines) was based on the average tumour absorbed doses for each patient (filled markers). The agreement for individual tumours is also indicated (open markers). The relative difference refers to the absorbed dose estimated from ^68^Ga-PET over the absorbed dose estimated through peri-therapeutic imaging

Figure 5 shows results of the Bland–Altman analysis of the agreement between tumour absorbed doses estimated from ^68^Ga PET images and serial peri-therapeutic ^177^Lu-imaging. On average, the ^68^Ga-based estimates obtained was 11% higher than the ^177^Lu absorbed doses measured at therapy, with a 95% coverage interval of − 65% to 248%. There were no discernible patterns associated with G1 or G2 NETs.

## Discussion

In this study, we have investigated the relationship between uptakes of [^68^Ga]Ga-DOTA-TATE quantified in PET images, and uptakes and absorbed doses to tumours and organs during subsequent treatment with [^177^Lu]Lu-DOTA-TATE for NETs. In summary, for tumours we see a significant (*p* < 0.05), moderately strong (*r* = 0.71), relationship across patients between the activity concentration from ^68^Ga-PET images and the absorbed dose from ^177^Lu-PRRT. A stronger relationship is seen with respect to the ^177^Lu activity concentration from SPECT images 24 h after injection. On an individual level, the ability to predict the ^177^Lu absorbed dose to tumours based solely on a ^68^Ga-PET image is limited, with a 95% coverage interval of − 65% to 248%.

The use of ^68^Ga-SSTR-PET for correlation with outcome and prognosis of NETs has been investigated both in general and with respect to ^177^Lu-PRRT [5–10]. However, the connection between ^68^Ga-SSTR-PET uptakes and absorbed doses during therapy is less studied [11]. Even if absorbed dose is not a direct measure of treatment outcome and toxicity, it is an established parameter in other forms of radiotherapy and is being gradually better established also for radionuclide therapy [35, 42, 43]. Hence, we believe that an increased understanding of relationships between ^68^Ga-SSTR imaging and absorbed doses in ^177^Lu-PRRT fills an important gap.

A fundamental difficulty for quantitative interpretation of pre-therapeutic ^68^Ga-SSTR-PET with respect to the absorbed doses delivered during ^177^Lu-PRRT lies in the different half-lives of ^68^Ga and ^177^Lu [44] (6.6 d versus 68 min [28, 29]). ^68^Ga-SSTR-PET is typically performed 1 h p.i. [4] while therapy with [^177^Lu]Lu-DOTA-TATE extends over several days or weeks [39]. So although the ligand is identical, the time scales of the processes exploited with ^68^Ga imaging and ^177^Lu therapy are markedly different, limiting the accuracy for prediction of the time-integrated activity and absorbed dose [45]. There are also other factors that differ between the ^68^Ga-SSTR-PET and ^177^Lu-PRRT, such as the method of administration (bolus versus extended infusion), and the fraction of the peptides that are radiolabelled which differs by nearly three orders of magnitude. At the same time, ^68^Ga-SSTR-PET imaging is today clinically used as part of the patient-selection process for ^177^Lu-PRRT, and hence, to some extent, a correlation is implicitly assumed.

For tumours, the strengths of the obtained correlations between uptakes of [^68^Ga]Ga-DOTA-TATE and absorbed doses in ^177^Lu-PRRT are on par with those reported previously for NET and meningioma [11, 13], and higher than those reported for satoreotide tetraxetan [12]. Comparison between uptakes in [^68^Ga]Ga-PSMA-11-PET and absorbed dose in therapy with ^177^Lu-PSMA-617 have also shown similar correlations [14]. Importantly however, from such correlations on a group level, it cannot be directly inferred that the therapeutic absorbed doses can be predicted for the individual patient. Based on the presented approach for prediction, using the ^68^Ga-PET activity concentration combined with population-based effective half-lives for [^177^Lu]Lu-DOTA-TATE for NETs, only rough estimates of the absorbed doses in the upcoming therapy are obtained (Fig. 5). Personalized treatment planning based on ^68^Ga-PET imaging will thus require more elaborate approaches, such as the inclusion of pharmacokinetic modelling [46].

The poor agreement between absorbed dose estimates (Fig. 5) can partly be theoretically explained by the combination of a protracted therapeutic delivery and a measurement at 1 h p.i. [45]. As such, considerable dispersion is expected. However, in principle, the accuracy of a measurement method needs to be considered in relation to the requirements for the application, and the results in Fig. 5 could then still be informative in cases when only a rough estimate is necessary. Apart from mathematical and biological considerations, different absorbed dose calculation methods are also used for the PET-based estimation compared to the peri-therapeutic dosimetry. However, the benefit of full Monte Carlo simulations compared with using local energy-deposition is typically small for ^177^Lu [39, 47] and is not expected to be the major reason for the disagreement between the estimated values.

Among the organs, only spleen exhibits a significant correlation between the uptake of [^68^Ga]Ga-DOTA-TATE and the absorbed dose in ^177^Lu-PRRT (Fig. 2). For kidneys, considered the primary organ-at-risk for ^177^Lu-PRRT, we see no significant relationship, one possible reason being the co-administration of renal protective amino acids for [^177^Lu]Lu-DOTA-TATE. For liver parenchyma, the estimation of the activity concentration suffers from practical challenges for VOI definition. Although small VOIs have been applied there is a risk that tumour may have been included, both due to spillover from adjacent tumours in the images and due to microscopic disease. Whether or not a patient is on treatment with long-acting SSA has, in previous publications, been observed to affect the liver uptake of [^68^Ga]Ga-DOTA-TATE and only to a lesser degree the tumour uptake [20]. According to the same authors variable time intervals from the last SSA injection did not affect uptake. It is therefore unlikely that this factor contributed to the dispersion in data for the liver and the tumour-to-liver ratio.

The stronger correlations obtained between the ^68^Ga and ^177^Lu activity concentrations, compared to the ^177^Lu absorbed dose (Table 1) were expected. Absorbed dose depends on a combination of initial activity uptake and excretion, while the activity concentration measured in ^68^Ga-PET at 1 h almost exclusively reflects the initial activity uptake. The uptake measured in ^177^Lu-SPECT at 24 h is less affected by the excretion than the absorbed dose is, which reduces the variability relative to the activity concentration at 1 h, measured in ^68^Ga-PET.

Of interest, our results provide no support for using different types of normalization of the ^68^Ga activity concentration to improve the relationship to absorbed dose in ^177^Lu-PRRT, neither with respect to normalization to body weight, i.e. calculation of SUV, nor with respect to a reference tissue. In this study, SUVs were calculated according to clinical practice, with no PVC applied, which may in part affect the correlations obtained. However, in relation to the ^177^Lu absorbed dose, there is no theoretical reason to normalize the activity concentration to body weight. Even if the body size, as an indirect measure of the plasma volume, may affect the activity uptake, this will act the same for diagnostics and therapy. Normalization to a reference tissue can in principle be motivated to cancel differences between receptor-bound activity and activity in blood in different patients. However, in our data such normalizations only increase the dispersion. The practical difficulties associated with the estimation of activity concentration or SUV in blood or liver parenchyma from ^68^Ga-PET images need to be emphasized. In a static ^68^Ga-PET image, blood SUV is associated with large uncertainties as it requires the measurement of low activity concentrations, which, in addition to the associated statistical variation, puts great demands on the accuracy of compensations for scattered and random coincidences. Thus, we believe that from both a theoretical and a practical point of view, it is preferable to study the AC/IA directly rather than normalized variants thereof.

For the correlation analyses for kidneys, the sensitivity to individual data points, as revealed by the leave-one-out analysis, should be noted (Fig. 2 and Table 1). The correlations obtained are largely governed by one or two data points rather than reflecting a general trend, and the significant correlations should hence be interpreted cautiously. Similar instability was not found for tumours.

The analysis of tumour data is more complex than for organs because of the varying number of tumours per patient, for which independence cannot be assumed. For this reason, the problem of finding a relationship between the uptakes in ^68^Ga-PET and the therapy is separated into two questions: 1) whether there is a relationship between patients when regarding the mean values for the tumours within each patient and 2) whether there is a relationship for the separate tumours within patients, following the methodology presented by Bland and Altman [40, 41]. Regarding the inter-patient analysis, a moderate correlation is obtained for the ^177^Lu-AD/IA as a function of ^68^Ga-AC/IA, while a stronger correlation is obtained for the ^177^Lu-AC/IA. This indicates that there is a group-level relationship between the uptake in ^68^Ga-PET and the ^177^Lu absorbed dose. The intra-patient analysis shows similar results, where the relationship is weaker for ^177^Lu-AD/IA than for ^177^Lu-AC/IA. This indicates that there is a correlation also within individual patients, i.e. on average a high ^68^Ga uptake for a separate tumour also corresponds to a high absorbed dose in subsequent ^177^Lu-PRRT. The two analyses are complementary, and it is concluded that there are statistically significant, but moderately strong, correlations both intra- and inter-patient.

Two important limitations of this study are the low number of included patients and the relatively permissive inclusion criterion of a [^68^Ga]Ga-DOTA-TATE PET performed up to 20 weeks prior to PRRT. The patient population was, however, one of well-differentiated NET with a low Ki67-index, i.e. the likelihood of significant change in tumour volume over the given time interval is small. Furthermore, the actual median time from PET imaging to PRRT was 5 weeks, further reducing such a potential confounder. The dosimetry methods used in this study have been extensively validated in previous papers [27, 30, 31]. In principle, however, image-based dosimetry based on SPECT-only imaging would be preferable to the hybrid method used. Furthermore, the employed cutoff volume of 5 mL is of concern, partly because it reduces the number of included tumours, but also because it systematically excludes tumours with a certain characteristic which could theoretically lead to biased results. However, the uncertainties associated with estimated volumes (Fig. 1) and activity concentrations of structures with dimensions close to the system spatial resolution are well known [31]. Thus, excluding the smallest tumours was considered necessary not to contaminate the results.

In summary, we find that there is a statistical relationship between tumour uptake at [^68^Ga]Ga-DOTA-TATE PET and absorbed dose to tumours in subsequent ^177^Lu-PRRT, but that this association is moderate at best. Given that previous studies have shown correlations of approximately the same strength, methodological differences notwithstanding, we believe that these moderate correlations reflect the actual strength of the relationship, rather than being a result of measurement uncertainties. Furthermore, we find no, or unstable, relationships for organs, except for spleen. Thus, at the group level there are relevant relationships between the uptake in [^68^Ga]Ga-DOTA-TATE PET and the upcoming ^177^Lu-PRRT. However, to be able to practically use [^68^Ga]Ga-DOTA-TATE PET for absorbed dose planning at the individual level, more complex models are needed that take patient-specific factors into account, beyond simple univariate analyses.

## Conclusion

On a group level, a higher tumour uptake of [^68^Ga]Ga-DOTA-TATE as measured from PET images 1 h p.i. is associated with higher absorbed doses in subsequent therapy with [^177^Lu]Lu-DOTA-TATE. On an individual level, the predictive power of absorbed dose estimates is limited. Correlations are not improved by using ^68^Ga SUV or normalized SUVs compared with using activity concentration per injected activity.

### Supplementary Information

**Additional file 1**: Volumes, SUVs, decay-corrected activity concentrations for ^68^Ga and ^177^Lu, and ^177^Lu absorbed doses for tumours and organs considered in this paper.

## Figures and Tables

**Fig. 6:**
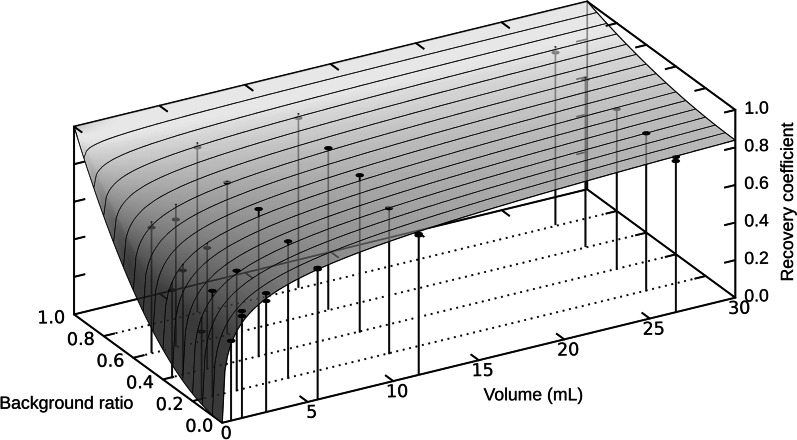
Measured and fitted recovery coefficient for ^68^Ga-PET as a function of sphere volume and background-to-object ratio

## Data Availability

All data generated or analysed during this study are included in this published article and its Additional file 1.

## Appendix

Figure 6 shows the RCs for ^68^Ga-PET derived from phantom data, and the in the fit of Eq. 3 parametrized in terms of volume and background-to-object ratio.

Tumour activity quantification in patient PET images was made in a sequence of steps, developed to mitigate the effects of the contracted VOIs obtained from image segmentation. Generally, an erroneous image segmentation has two, positively correlated, effects on the estimated activity concentration [48]: a) the erroneous VOI affects the apparent activity concentration, and b) the error in the estimated volume propagates to an error in the estimated RC. Thus, for the patient PET images, if not corrected for, the erroneously small VOIs applied to the high-signal tumours would both lead to an increased apparent concentration and a falsely low RC (Eq. 3), together yielding a positive error in the estimated activity concentration. To mitigate both effects (a) and (b), the RC in Eq. 3 was recalculated to be applicable to the VOI with volume $V_{{\text{S}}}$.

This recalculation was made based on the combination of Eq. 1 and 3, and digital phantom-based modelling. For each tumour, $V_{{\text{p}}}$ was calculated (Eq. 1) and a voxelized sphere constructed. This was placed in a background according to the estimated $\eta$. Effects of limited spatial resolution were modelled as a convolution with a Gaussian point-spread function (PSF). The FWHM of this PSF was adapted to yield a recovery according to Eq. 3 using a minimization of the squared difference with a golden section search [49]. A spherical VOI was then defined, with volume $V_{{\text{s}}}$ obtained from the Fourier surface segmentation, and an updated recovery coefficient, $R^{\prime } \left( {V_{{\text{s}}} ,\eta } \right)$, calculated. For determination of $\eta$ in patient images, the background concentration was estimated as the mean activity concentration in a 3-voxel thick shell surrounding the tumour VOI, with a 2 cm spacing between the shell and the outer VOI edge in all directions. For a given volume, the values of $R^{\prime }$ and $\eta$ were determined in an iterative fashion, where: i) the recovery was calculated for the current estimate of $\eta$, ii) $R^{\prime }$ was applied to obtain a corrected VOI activity concentration, iii) an updated value of $\eta$ was calculated. Steps (i)-(iii) were repeated until $\eta$ had stabilized. At application for tumour activity quantification in patient images, the factor $R^{\prime } \left( {V_{{\text{s}}} ,\eta } \right)$ was applied for RC.

## Abbreviations

AC: Activity concentration
AD: Absorbed dose
CT: Computed tomography
FWHM: Full width at half maximum
IA: Injected activity
NET: Neuroendocrine tumour
PET: Positron emission tomography
p.i.: Post-injection
PRRT: Peptide receptor radionuclide therapy
PSF: Point spread function
PVC: Partial-volume correction
RC: Recovery coefficient
ROI: Region of interest
SPECT: Single photon emission computed tomography
SSA: Somatostatin analogue
SSTR: Somatostatin receptor
SUV: Standardized uptake value
VOI: Volume of interest
